# Resection of rectal cancer resembling submucosal tumor that was preoperatively diagnosed with endoscopic ultrasound-guided biopsy

**DOI:** 10.1186/s40792-017-0362-7

**Published:** 2017-07-26

**Authors:** Akimitsu Tanio, Hiroaki Saito, Keigo Ashida, Shouichi Urushibara, Manabu Yamamoto, Naruo Tokuyasu, Teruhisa Sakamoto, Soichiro Honjo, Yoshihiko Maeta, Yoshiyuki Fujiwara

**Affiliations:** Division of Surgical Oncology, Department of Surgery, School of Medicine, Tottori University Faculty of Medicine, 36-1 Nishi-cho, Yonago, 683-8504 Japan

**Keywords:** Colorectal cancer, Endoscopic ultrasound-guided fine needle aspiration biopsy, Submucosal tumor

## Abstract

**Background:**

Colorectal cancer (CRC) resembling submucosal tumor (SMT; CRC/SMT) is very rare. Because its biopsy is challenging, accurate preoperative diagnosis is also very rare.

**Case presentation:**

A 55-year-old woman with a high serum carcinoembryonic antigen level underwent a computed tomography colonoscopy, which showed extrinsic rectum compression. A coronal magnetic resonance image showed a 4-cm low-intensity tumor between her rectum and sacrum. Endoscopic ultrasound (EUS) showed a 30-mm low-echoic lesion originating from the rectum. Pathological examination of specimen obtained with EUS-guided fine-needle aspiration biopsy (EUS-FNAB) revealed adenocarcinoma. Immunohistochemical staining showed the tumor to be positive for both CK20 and CDX2 and negative for CK7, indicating that it was a rectal cancer. We performed a laparoscopy-assisted low-anterior resection with dissection of the regional lymph nodes after eight chemotherapy cycles. Macroscopically, tumor was completely covered by normal rectal mucosa, but showed a 2-mm bulge on the mucosa. Histological examination revealed a moderately differentiated adenocarcinoma, mainly located at the subserosal layer and severely invaded to lymphatic and blood vessels. The mucosal layer was not exposed to the cancer components, and her postoperative course was uneventful.

**Conclusion:**

EUS-FNAB was useful in preoperative accurate diagnosis of this very rare tumor. We also review the literature and discuss CRC/SMT.

## Background

Submucosal tumor (SMT) usually arises from tissue in the wall of digestive tract, and its surface is therefore covered with normal mucosa in most cases. In contrast, gastrointestinal (GI) carcinomas arise from the epithelium, and most of their mucosal surfaces typically consist of cancerous tissue. SMT-like growth is an unusual presentation for GI carcinomas, especially in colorectal cancer (CRC). We herein report a rare case of rectal cancer resembling SMT (CRC/SMT) in which endoscopic ultrasound-guided fine needle aspiration biopsy (EUS-FNAB) was useful in accurate preoperative diagnosis. We also review the literature and discuss CRC/SMT.

## Case presentation

**Fig. 1:**
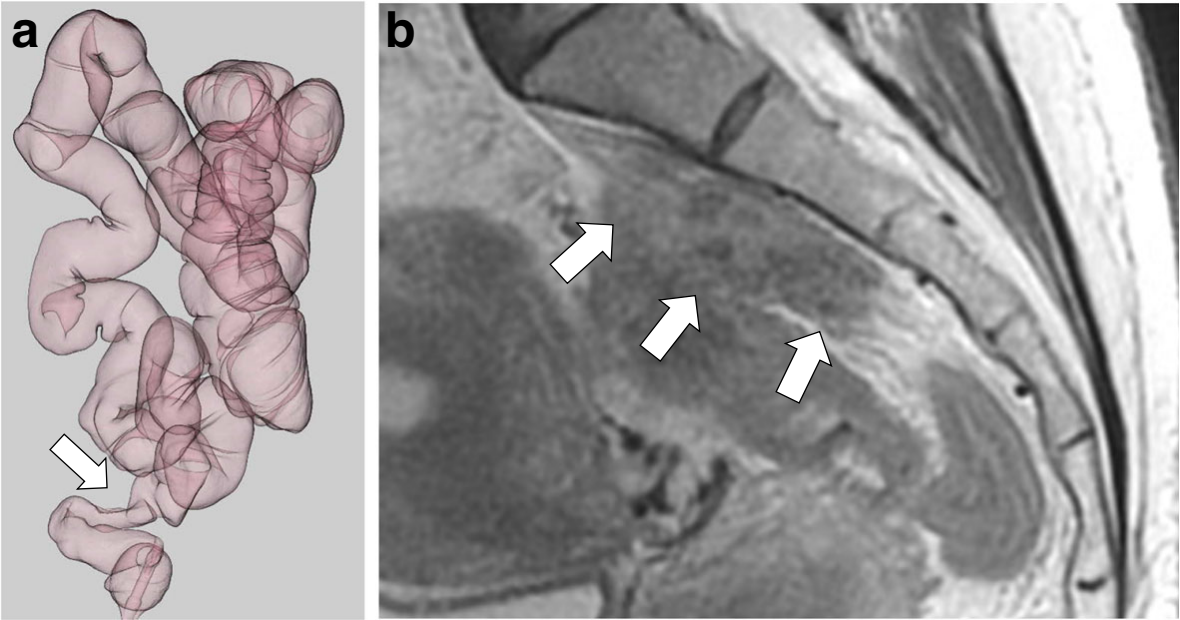
**a** CT colonoscopy revealed extrinsic compression of rectum. **b** Coronal magnetic resonance imaging (MRI) showed 4-cm low-intensity tumor between rectum and sacrum

**Fig. 2:**
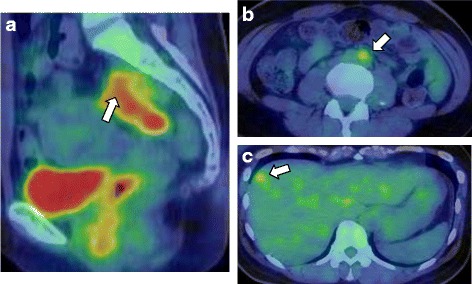
The 8-FDG positron emission tomography (PET) and CT scans indicate high 18-FDG uptake in (**a**) the primary tumor (SUVmax: 3.34); (**b**) enlarged para-aortic lymph node (SUVmax: 2.58); and (**c**) anterosuperior segment of right hepatic lobe (SUVmax: 2.74)

**Fig. 3:**
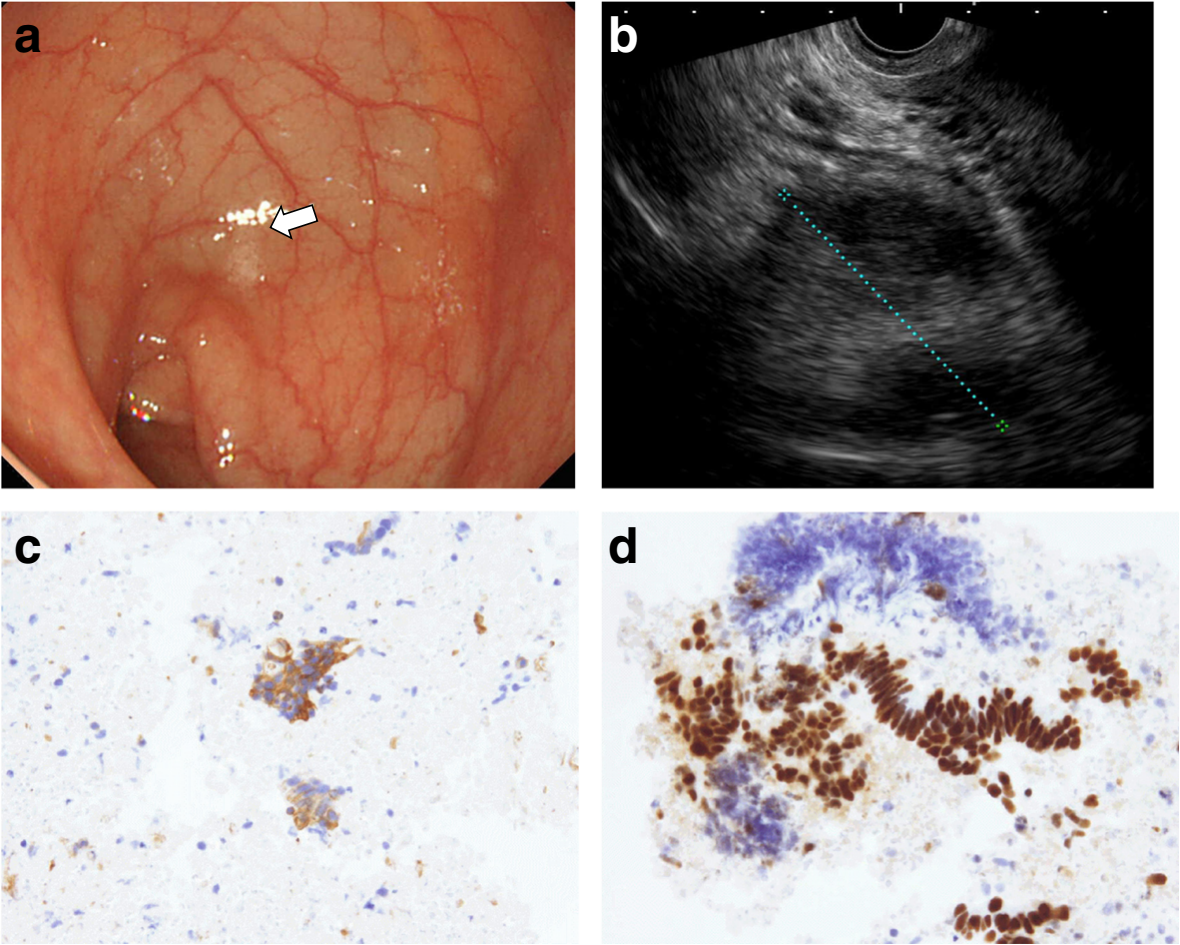
**a** Colonoscopy also showed extrinsic compression of rectum, but no change in rectal mucosa. **b** Endoscopic ultrasound (EUS) showed a 30-mm low echoic lesion originating from rectum. Immunohistochemical staining of biopsy specimen obtained by EUS-FNAB showed that tumor was positive for both (**c**) CK20 and (**d**) CDX2

**Fig. 4:**
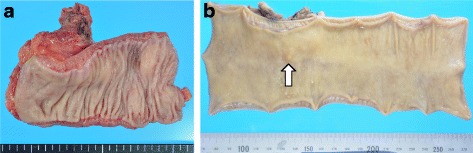
**a** Macroscopically, the tumor was completely covered by normal rectal mucosa. **b** The formalin-fixed resected specimen showed a 2-mm bulge above the mucosa level

**Fig. 5:**
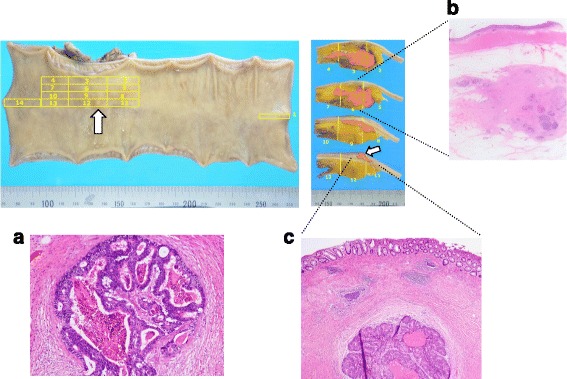
**a** Histologically, the tumor was composed of moderately differentiated adenocarcinoma. **b** The carcinoma was mainly located at subserosa and had severely invaded to lymphatic and blood vessels. **c** Adenocarcinoma was observed in the submucosal layer and the mucosal surface showed no exposure of the cancer component

An asymptomatic 55-year-old woman underwent a detailed GI tract examination, as she was found to have high serum carcinoembryonic antigen (CEA, 26.6 ng/ml) at a local hospital. She had no event in her past or family history, especially for colorectal neoplasm or other malignancy. Physical examination and blood chemical examination were within normal ranges except for high serum CEA level. Computed tomography (CT) colonoscopy revealed extrinsic rectum compression (Fig. 1a). A coronal magnetic resonance image (MRI) showed a 4-cm low-intensity tumor between the rectum and sacrum (Fig. 1b); the patient was referred to our hospital for further evaluation of this tumor. The 18-FDG positron emission tomography (PET) and CT scans indicated high 18-FDG uptake in the primary tumor (SUVmax: 3.34; Fig. 2a), with enlarged para-aortic lymph node (SUVmax: 2.58; Fig. 2b) and anterosuperior segment of right hepatic lobe (SUVmax: 2.74; Fig. 2c). Colonoscopy showed extrinsic rectum compression, but no change in her rectal mucosa (Fig. 3a). Pathological examination of rectum biopsy specimen showed normal rectal mucosa. Endoscopic ultrasound (EUS) showed a 30-mm, low-echoic lesion originating from the rectum (Fig. 3b). Pathological examination of biopsied specimen obtained with EUS-guided fine-needle aspiration biopsy (EUS-FNAB) revealed adenocarcinoma. Immunohistochemical staining showed that the specimen was positive for both cytokeratin 20 (CK20; Fig. 3c) and caudal-type homeobox 2 (CDX2; Fig. 3d) and negative for cytokeratin-7 (CK7), which indicated that it had originated from rectal cancer. We planned to perform low-anterior resection with dissection of the regional lymph nodes and an enlarged para-aortic lymph node under the diagnosis of rectal cancer and possible metastasis to liver and para-aortic lymph node, followed by resection of possible liver metastasis after postoperative chemotherapy. Because of a possibility that tumor had invaded to the sacrum, however, we performed neoadjuvant chemotherapy with capecitabine and oxaliplatin. The tumor shrank by 38% after 8 courses of chemotherapy, and we performed a laparoscopy-assisted low-anterior resection with dissection of the regional lymph nodes as planned. Although we confirmed direct invasion of the tumor to hypogastric nerve during the surgery, the tumor did not invade to the sacrum. An enlarged para-aortic lymph node was also removed. Macroscopically, the tumor was completely covered by normal rectal mucosa (Fig. 4a), although there was a 2-mm bulge of elevated lesion on the mucosa (Fig. 4b). Histologically, the tumor was composed from moderately differentiated adenocarcinoma (Fig. 5a) and was mainly located at subserosal region with severe invasion to lymphatic and blood vessels (Fig. 5b). However, the mucosal surface showed no exposure of the cancer component (Fig. 5c). Metastases were found in two lymph nodes of mesorectum (#251) and in one lymph node along the superior rectal artery (#252), although there was no metastasis in the para-aortic lymph node dissected. The postoperative course was uneventful. The patient is currently undergoing chemotherapy for liver metastasis in the outpatient clinic of our hospital.

## Discussion

Carcinomas of the digestive tract arise from the mucosal epithelium; most of their mucosal surfaces consist of cancerous tissues. Although cancer cells then infiltrate both horizontally and vertically, the dominant direction depends on the nature of the cancer cells. Some intestinal cancers dominantly infiltrate in a vertical direction without massive invasion along the horizontal plane. As a result, the internal intestinal surfaces over these tumors are mostly covered with normal mucosa, and therefore manifest as SMTs. This type of tumor is sometimes called a carcinoma resembling an SMT; and although it is extremely rare, has been reported in the esophagus [1], stomach [2], and colon and rectum [3, 4]. A report that reviewed 70 reported cases of CRC/SMT found it to be characterized by small tumor size, high rates of poorly differentiated adenocarcinoma or mucinous adenocarcinoma, and invasiveness (including high rates of lymph node metastasis and lymphatic vessel invasion) [4].

Two points make our case different from previously reported CRC/SMT. Firstly, the tumor was completely covered by normal rectal mucosa in our case. Most tumors reported previously showed at least faint mucosal changes, such as erosion and ulceration, which are microscopically consistent with exposure of carcinoma cells. Because most of the cancer on our case was located subserosally with no exposure of cancer cells on mucosal surface, metastatic rectal cancer was considered. In this regard, immunohistochemical staining of the biopsied specimen showed the tumor was positive for CK20 and negative for CK7, indicating the strong possibility that our patient had primary rectal cancer. To the best of our knowledge, only four cases (including the present case) have been reported of CRC/SMT that were completely covered with normal rectal mucosa (Table 1) [5–7]. Why no cancer cells were exposed on the mucosal layer remains unclear. Cancer may arise from ectopic mucosal cells in the rectal wall. Further investigations are required to show the detailed mechanism.

**Table 1:** Reported cases of colorectal cancer resembling SMT in which tumor was completely covered with normal colorectal mucosa

Case	Year	Age (years)	Gender	Location^a^	Size (cm)	Endoscopic findings	Depth of invasion^b^	Histologic diagnosis of biopsy specimen	Histologic diagnosis of resected specimen^c^	Lymph node metastasis	Distant metastasis	Reference
1	2005	57	M	Rb	10	None	A	Unknown	Muc	Absent	Unknown	5
2	2011	82	M	S	8	None	SS	Normal mucosa	Muc	Absent	Absent	6
3	2014	79	M	Ra	2.6	Erosion	SE	Not performed	Mod	Absent	Absent	7
4	2017	55	F	RS	4	None	SS	Normal mucosa	Mod	Present	Liver metastasis	Our case

^a^Location. *Ra* upper rectum, *Rb* lower rectum, *RS* rectosigmoid, *S* sigmoid colon

^b^Depth of invasion. *A* adventitia, *SE* serosa, *SS* subserosa

^c^Histologic diagnosis of resected specimen. *Mod* moderately differentiated adenocarcinoma, *Muc* mucinous carcinoma

Secondly, our case clearly showed that EUS-FNAB is useful for accurate preoperative diagnosis of CRC/SMT. EUS-FNAB was recently shown to be useful in accurate preoperative diagnosis of gastrointestinal stromal tumor (GIST) [8] and pancreatic cancer [9]. Previous reports have shown the difficulty of preoperative CRC/SMT diagnosis, because of the difficulty of obtaining biopsy specimens. Therefore, EUS-FNAB should be considered to make accurate preoperative SMT diagnoses in colon and rectum. To our knowledge, our case is the first to use EUS-FNAB for an accurate preoperative diagnosis of CRC/SMT.

## Conclusion

We should keep in mind that colorectal cancer can present with SMT-like growth. Furthermore, EUS-FNAB is useful for preoperative accurate diagnosis of this rare colorectal cancer.
